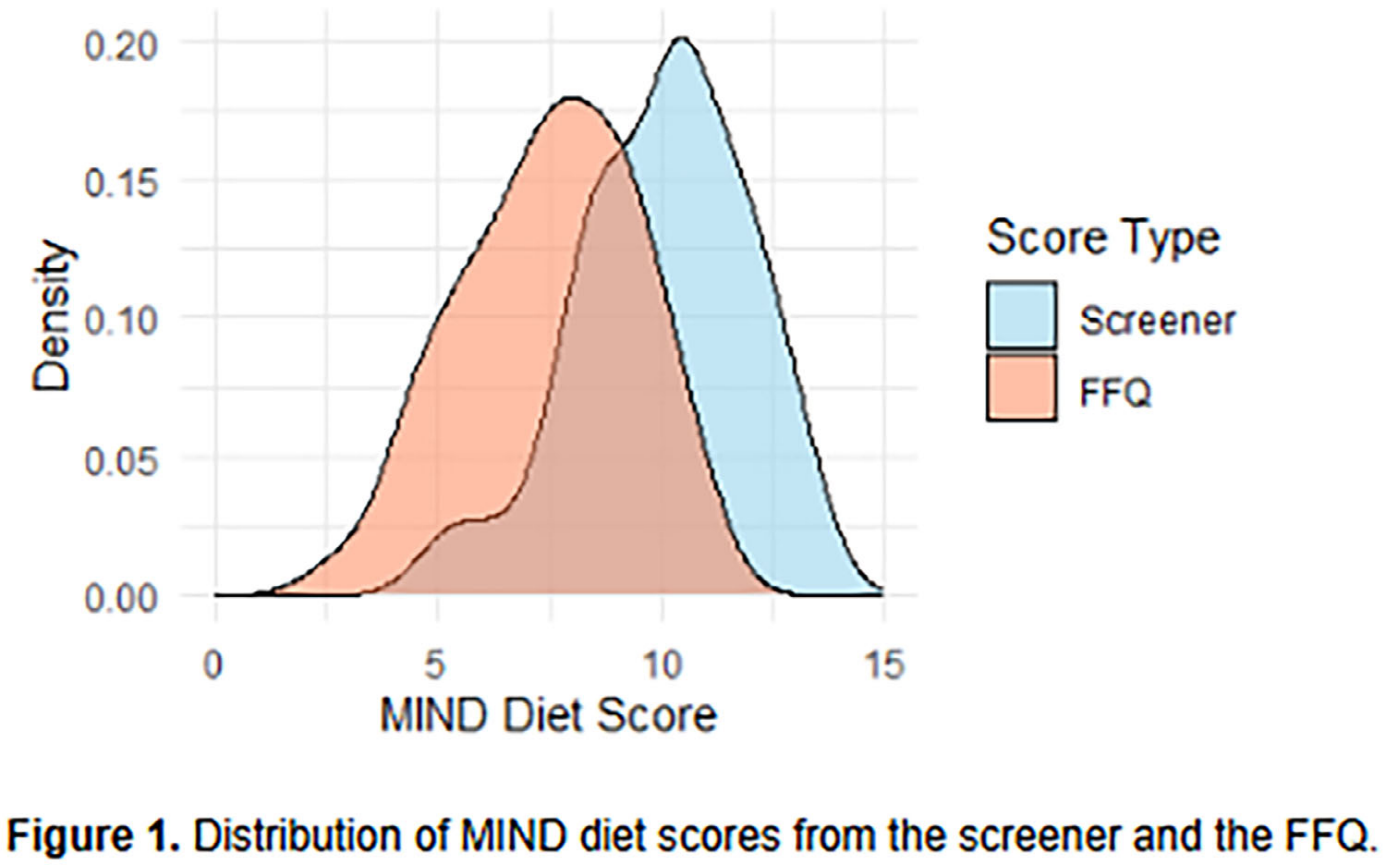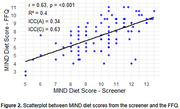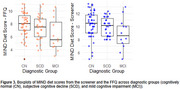# Correspondence Between the MIND Diet Adherence Screener and Food Frequency Questionnaire

**DOI:** 10.1002/alz70860_105690

**Published:** 2025-12-23

**Authors:** Desarae A. Dempsey, Puja Agarwal, Shane Fernandez, Jared R. Brosch, Michelle Quirke, Yolanda Graham‐Dotson, Sujuan Gao, Daniel Clark, Liana G. Apostolova, David G. Clark, Martin R. Farlow, Sunu Mathew, Frederick Unverzagt, Sophia Wang, Edgar Diaz, Leyla Schimmel, Robin French, Colette Blach, Rima F. Kaddurah‐Daouk, Andrew J. Saykin, Shannon L Risacher, Alzheimer Gut Microbiome Project Consortium

**Affiliations:** ^1^ Indiana Alzheimer's Disease Research Center, Indianapolis, IN, USA; ^2^ Indiana University School of Medicine, Indianapolis, IN, USA; ^3^ Rush Alzheimer's Disease Center, Rush University Medical Center, Chicago, IL, USA; ^4^ Centre for Precision Health, Edith Cowan University, Joondalup, Western Australia, Australia; ^5^ Collaborative Genomics and Translation Group, School of Medical and Health Sciences, Edith Cowan University, Joondalup, Western Australia, Australia; ^6^ Indiana Alzheimer's Disease Research Center, Indiana University School of Medicine, Indianapolis, IN, USA; ^7^ Indiana University Center for Aging Research, Indianapolis, IN, USA; ^8^ Duke University, Durham, NC, USA; ^9^ Indiana University School of Medicine, Department of Radiology and Imaging Sciences, Indianapolis, IN, USA

## Abstract

**Background:**

The Mediterranean‐DASH Intervention for Neurodegenerative Delay (MIND) diet has been associated with cognitive benefits and reduced risk of Alzheimer's disease. Adherence is typically assessed using comprehensive but time‐consuming food frequency questionnaires (FFQs). We examined concurrent validity between a brief MIND diet screener and a more extensive FFQ.

**Methods:**

94 participants (51 cognitively normal (CN), 31 subjective cognitive decline (SCD), 12 mild cognitive impairment (MCI)) from the Indiana Alzheimer's Disease Research Center (IADRC) who participated in the Alzheimer's Gut Microbiome Project (AGMP) completed both the self‐reported 15‐item MIND screener and computerized Vioscreen FFQ. For both measures, we used the same cutoff criteria to assign values of 0, 0.5, or 1 corresponding to low, medium, and high intake for the ‘healthy’ food groups and reverse correspondence for the ‘unhealthy’ food groups, which were then summed to generate a total MIND diet score (0‐15) with higher scores indicating greater adherence. Agreement between the two methods was assessed using Pearson correlation, intraclass correlation coefficient (ICC) for absolute agreement and consistency, and a tertile‐based cross‐classification. ANOVA was used to test differences in MIND scores between diagnostic groups, adjusting for age, sex, and education.

**Results:**

The mean MIND diet score from the FFQ was 7.49 (range: 2.5‐11), and from the screener was 10.05 (range: 5‐13.5), with a mean 2.56‐point difference showing consistently higher scores on the screener (Figure 1). The screener demonstrated moderate correlation with the FFQ score (*r* = 0.63, *p* <0.001, R2=0.40). Absolute agreement was low (ICC=0.34), while consistency was moderate (ICC=0.64) (Figure 2). In cross‐classification, 19.15% of individuals were classified into disparate tertiles. A significant difference was observed between CN and MCI groups using both methods, but only the screener‐derived score remained marginally significant after adjustments (*p* = 0.05) (Figure 3).

**Conclusions:**

The MIND screener shows moderate correlation and consistency with the FFQ, with participants systematically reporting higher scores on the screener, indicating overestimation of their MIND diet score. While the screener does not capture detailed or food item specific dietary variations assessed by the FFQ, it is a valid tool for rapid estimation of MIND diet score and may be useful in research and clinical practice.